# Supplementary material for: Chronic presence of blood circulating anti-NMDAR1 autoantibodies impairs cognitive function in mice
Source: PLoS One. 2021 Sep 2;16(9):e0256972. doi: 10.1371/journal.pone.0256972 (PMC8412244; doi:10.1371/journal.pone.0256972)
Supplement: Attachment — Submitted filename: Responses to Critiques.docx [file pone.0256972.s004.docx]

**Responses to Critiques**

I appreciate a thorough review, enthusiasm, and constructive suggestions from both the Editor and the reviewers. Accordingly, I have incorporated these suggestions and critiques into a revised manuscript. I am now addressing all the concerns the reviewers raised.

Reviewer 1

*1. Fig 2 showed that antibodies generated in mice after immunization with P2 peptide bind to the naive NMDAR1 protein that are expressed in HEK203 cells by transfection of cDNA. However, it is unclear whether the antibodies are specific to NR1 protein and no other cross linked in this study. It is imperative to confirm that auto-antibody recognize only NR1 protein and no other major bands on the Western blotting. If other Mol. Weight bands did appear on the blot, I do not think auto-antibody against NR1 is “sufficient” to impair behavioral phenotypes.*

A). The anti-NMDAR1 autoantibodies are produced by active immunization of mouse NMDAR1 peptide P2 with the CFA. The anti-NMDAR1 autoantibodies recognize NMDAR1 proteins that have a typical expression pattern in mouse hippocampus. Mice immunized only with the CFA do not produce anti-NMDAR1 autoantibodies and do not have staining in mouse hippocampus.

B). The specificity of the anti-NMDAR1 autoantibodies was further confirmed by peptide blocking experiment in Fig1D. Only the mouse NMDAR1 P2 peptide can block the binding of the autoantibodies to the NMDAR1 proteins in mouse hippocampus, whereas the NMDAR1 P1 peptide and BSA have no blocking activity at all.

C). We purchased the BIOCHIP from Euroimmun to further validate the binding of the anti-NMDAR1 autoantibodies to native NMDAR1 proteins expressed on HEK293 cells transfected with the NMDAR1 gene only. This cell-based assay is a standard diagnostic test to detect anti-NMDAR1 autoantibodies for anti-NMDAR1 encephalitis. We further conducted co-immunocytochemical staining to demonstrate a complete co-localization between known human anti-NMDAR1 autoantibody and the mouse anti-NMDAR1 autoantibodies against the P2 peptide (Fig 2B). Unlike Western blot for detection of denatured NMDAR1 proteins, the co-immunocytochemical analysis is superior in specificity.

D). In One-Step assay (Fig 8B), the anti-NMDAR1 autoantibodies were incubated with either the NMDAR1 P2 peptide tagged with GFP or NMDAR1-GFP fusion proteins that were produced and purified from *E coli* using 6His purification system. The anti-NMDAR1autonatibodies specifically bind both the P2-GFP and NMDAR1-GFP proteins, but not GFP proteins.

*2. The authors claimed in Abstract and main text that presence of anti-NMDAR1 auto-antibody is sufficient to specifically impair T-maze spontaneous alternation. However, the first cohort of mice in a pilot study, they were also clearly impaired in the fear memory extinction. I do not think T-maze is “specifically” impaired. Please remove this word.*

We removed “specifically’ from the manuscript. However, the fear conditioning phenotypes in the small pilot mouse cohort cannot be replicated in the subsequent two large cohorts. These phenotypes are still questionable.

*3. No description about the consequence of using P1 peptide. Then why P1 was described in the Method? Please clarify.*

The P1 peptide was used as a control peptide for the P2 peptide blocking experiments to demonstrate the specificity of the anti-NMDAR1 autoantibodies in Fig 1D.

*4. T-maze alternation is defective although no immunohistological abnormality was detected in the staining in the striatum hippocampus and cortex in Fig 11. Medial PFC is also crucial for short-term memory in T-maze alternation. It is suggested to examine the integrity of mPFC.*

We agree with the reviewer that our next goal will be to examine whether we may find any reduction of NMDAR1 proteins in mPFC.

Reviewer 2

1) *The authors used P2 peptide derived from ligand-binding domain of mouse NMDAR1 protein for immunization. Why did authors select this peptide? Does this peptide sequence locate on the surface of the NMDAR1 subunit molecule? The crystal structure of the subunit was reported, thus the rationale of the selected sequence is necessary.*

We provided the rationale for the selection of the peptide antigens in Materials:

“Selection of the peptides was based on immunogenicity (<http://www.cbs.dtu.dk/services/NetMHCIIpan/>), solubility, and surface localization on NMDAR1 proteins”

2) *The quantification of the titer of the anti-NMDAR1 autoantibody has some problems. The evaluation using immunohistochemical analysis is semi-quantitative. The authors used differential staining intensities between hippocampal CA1 and corpus callosum with autoantibodies as shown in Fig. 1C and 1D. In the 2nd and 3rd experiments with large cohort, the immunization protocol is different from the 1st experiment with small cohort. Furthermore, in the 3rd cohort, the authors evaluate the presence of the autoantibodies with the different in vitro One-Step quick assay. Is there any consistency of the titers of autoantibodies between immunohistochemical method and in vitro method? If the authors used same evaluation method of the titers of autoantibody in the three cohorts, the authors could analyze the relationship between the titer of the autoantibodies and behavior phenotypes in each mouse.*

The immunization protocol is the same across all 3 mouse cohorts. The only difference is the change of Loss-of-Resistance (LOS) syringes that makes emulsification more effortless in the large mouse cohorts.

In the 3^rd^ cohort, we used One-Step Quick assay to screen the anti-NMDAR1 autoantibodies. The One-Step quick assay is 100% consistent with the IHC analysis. Since the One-Step assay uses GFP for detection, it was used as a qualitative test. The reviewer raises an important question as to whether T-maze phenotypes may correlate to the titers of the anti-NMDAR1 autoantibodies. We did not collect mouse blood immediately after T-maze test because we did not want blood collection to complicate subsequent behavioral tests. Since the levels of blood anti-NMDAR1 autoantibodies can change significantly, the titers of the later-collected anti-NMDAR1 autoantibodies cannot be used for such correlation analysis. In the future, we need a much large mouse cohort to specifically address this question.

3) *Did the autoantibody against the P2 peptide specifically recognize the NMDAR1 subunit in the mouse brain? The brain section derived from the NMDAR1-KO mouse is the ideal negative control. Or, do the autoantibodies detect only NMDAR1 subunit with western blot analysis?*

The specificity of the anti-NMDAR1 autoantibodies has been demonstrated in the peptide blocking experiments in Fig1D. The specificity of the anti-NMDAR1 autoantibodies has been addressed in the first question of the Reviewer 1.

Minor points
*1) In the “Materials and Methods”, the 2nd to 4th lines in the “Fear conditioning” section need to be corrected to easy understand.*

We clarified the test and added one more reference.

*2) In the “Results”, statistical analysis of effect size shown in the Fig 6C is d = 1.296, but in the text “effect size (d = 1.29)” . The description of (d = 1.30) is better.*

Corrected with d=1.296 in the text.

*3) In the Figure Legend of Figure1 (A), CTD is not shown in Fig.1.*

CTD was added.

*4) In the Figure Legend of the title of Figure 4, not only “Fear extinction”.*

“Fear extinction” was changed into “Fear Conditioning”.

*5) Fig. 2 and 8B, size markers are helpful.*

Bars were added.
